# Supplementary material for: Supra-threshold vibration applied to the foot soles enhances jump height under maximum effort
Source: PLoS One. 2022 Apr 6;17(4):e0266597. doi: 10.1371/journal.pone.0266597 (PMC8985973; doi:10.1371/journal.pone.0266597)
Supplement: Attachment — Submitted filename: Response to Reviewers.docx [file pone.0266597.s002.docx]

***We thank the editor and reviewers for the feedback on the manuscript. Here, we provide detailed responses to all the points. Please note that the review comments are provided in black font, and our responses are in blue font.***

1. Please ensure that your manuscript meets PLOS ONE's style requirements, including those for file naming.

Thank you for your advice. We revised the manuscript and file names according to PLOS ONE's style requirements.

2. Thank you for stating the following in the Competing Interests section:

JM, PP, SR, CR, YS, and JA are inventors of two patents (10-2019-0118121 and 10-2019-0170835), which were applied to Korean Intellectual Property Office by SNU R&DB Foundation. The active insole shoes used in this study were developed by the Samsung Electronics, which employs S.R., C.R., and Y.S. These shoes might be commercialized in the future.

Please confirm that this does not alter your adherence to all PLOS ONE policies on sharing data and materials, by including the following statement: "This does not alter our adherence to PLOS ONE policies on sharing data and materials.” (as detailed online in our guide for authors http://journals.plos.org/plosone/s/competing-interests). If there are restrictions on sharing of data and/or materials, please state these. Please note that we cannot proceed with consideration of your article until this information has been declared.

Please include your updated Competing Interests statement in your cover letter; we will change the online submission form on your behalf.

Two of the authors (CR and YS) changed their affiliations, and we updated the manuscript and Competing Interests section accordingly. We confirm that the situation clarified in the Competing Interests statement does not alter our adherence to PLOS ONE policies on sharing data and materials. Following the editor’s suggestion, we also included our updated Competing Interests statement in the cover letter.

3. We note that Figure 1 in your submission contain copyrighted images. All PLOS content is published under the Creative Commons Attribution License (CC BY 4.0), which means that the manuscript, images, and Supporting Information files will be freely available online, and any third party is permitted to access, download, copy, distribute, and use these materials in any way, even commercially, with proper attribution. For more information, see our copyright guidelines: http://journals.plos.org/plosone/s/licenses-and-copyright.

We require you to either (1) present written permission from the copyright holder to publish these figures specifically under the CC BY 4.0 license, or (2) remove the figures from your submission:

a. You may seek permission from the original copyright holder of Figure 1 to publish the content specifically under the CC BY 4.0 license.

We recommend that you contact the original copyright holder with the Content Permission Form (http://journals.plos.org/plosone/s/file?id=7c09/content-permission-form.pdf) and the following text:

“I request permission for the open-access journal PLOS ONE to publish XXX under the Creative Commons Attribution License (CCAL) CC BY 4.0 (http://creativecommons.org/licenses/by/4.0/). Please be aware that this license allows unrestricted use and distribution, even commercially, by third parties. Please reply and provide explicit written permission to publish XXX under a CC BY license and complete the attached form.”

Please upload the completed Content Permission Form or other proof of granted permissions as an "Other" file with your submission.

In the figure caption of the copyrighted figure, please include the following text: “Reprinted from [ref] under a CC BY license, with permission from [name of publisher], original copyright [original copyright year].”

b. If you are unable to obtain permission from the original copyright holder to publish these figures under the CC BY 4.0 license or if the copyright holder’s requirements are incompatible with the CC BY 4.0 license, please either i) remove the figure or ii) supply a replacement figure that complies with the CC BY 4.0 license. Please check copyright information on all replacement figures and update the figure caption with source information. If applicable, please specify in the figure caption text when a figure is similar but not identical to the original image and is therefore for illustrative purposes only.

We (the authors) took Figure 1 by ourselves; we are the original copyright holder. Considering the Editor’s concern and following the suggestion from a PLOS ONE journal staff, Rose Ann Joyce Sagun Puetes, we additionally made the commercial logo on the tongue of the shoe invisible. We now confirm that no copyrighted material is present in Figure 1.

4. We note that Figure 2 includes an image of a participant in the study].

As per the PLOS ONE policy (http://journals.plos.org/plosone/s/submission-guidelines#loc-human-subjects-research) on papers that include identifying, or potentially identifying, information, the individual(s) or parent(s)/guardian(s) must be informed of the terms of the PLOS open-access (CC-BY) license and provide specific permission for publication of these details under the terms of this license. Please download the Consent Form for Publication in a PLOS Journal (http://journals.plos.org/plosone/s/file?id=8ce6/plos-consent-form-english.pdf). The signed consent form should not be submitted with the manuscript, but should be securely filed in the individual's case notes. Please amend the methods section and ethics statement of the manuscript to explicitly state that the patient/participant has provided consent for publication: “The individual in this manuscript has given written informed consent (as outlined in PLOS consent form) to publish these case details”.

If you are unable to obtain consent from the subject of the photograph, you will need to remove the figure and any other textual identifying information or case descriptions for this individual.

Thank you for your advice. We received written informed consent (PLOS consent form) from the participant shown in Figure 2. We also added the statement about the participant’s consent for publication in the revised manuscript (lines 90-91).

5. Please review your reference list to ensure that it is complete and correct. If you have cited papers that have been retracted, please include the rationale for doing so in the manuscript text, or remove these references and replace them with relevant current references. Any changes to the reference list should be mentioned in the rebuttal letter that accompanies your revised manuscript. If you need to cite a retracted article, indicate the article’s retracted status in the References list and also include a citation and full reference for the retraction notice.

In the revision, we added a few references to address comments from the reviewers. We rechecked all the updated references and found that no reference has been retracted.

**Reviewer #1**

Dear author,

Line 62: citation needed.

Thank you for your suggestion. In the revision, we cited the reference showing that removed or reduced sensitivity decreases the motor performance (line 64).

Line 111-116: citation needed, & need to explain the procedure of markers placement.

That paragraph explains the equipment and methods we used in our own study. To clarify further, no marker set was applied because we did not record kinematics. Instead, we calculated vertical jump height (VJH) of each subject based on the impulse generated during jump motion and the subject’s body weight (BW).

Line 216-219: citation needed on the procedure filtering and smoothing.

In the revision, we added the reference for the procedure of EMG signal filtering and smoothing (line 221).

Line 223 - Results: How many t-test was conducted? It seems many paired t-test thus increase chances of Type 1 error. Need to rethink better options of analysis (e.g. 1 WAY RM ANOVA).

We understand that Type-I error could be inflated in determining the significance among three or more conditions and it should be corrected by the Bonferroni adjustment for pairwise comparison (MacDonald, P.L., & Gardner, R.C., *Edu. Psychol. Meas.* **60**, 735-754 (2019)). However, in this study, we aimed to compare outcome variables under “two conditions”: with vibration and without vibration. To recap our study, we performed one paired t-test to check the effect of vibration on the vertical jump height, and then, we performed another paired t-test to check the effect of vibration on the muscle activation level. Therefore, in the case of our study, the two independently performed t-tests do not affect the chance of Type-I error.

**Reviewer #2**

This paper presents the proof-of-concept of custom-built active vibrating insoles in shoes with mechanical vibration delivered to evaluate the change in the jump height and muscle activation.

Vertical jump height, body weight and impulse generated during jump motion were calculated.

For suggestion, it is worthwhile to mention why the amplitude of insole vibration applied was 130% of the sensory threshold. Eventhough this value is based on a previous study, are there any similarities or differences between this study and the previous research? Does this value based on the design of the product or necessary for the jump activity?

We appreciate reviewer’s comment and suggestion. As we explained in the manuscript, we chose the amplitude of the vibration as 130% of the threshold considering a previous study that demonstrated the efficacy of such vibration during a mildly difficult balancing task. However, this choice was not based on any similarity between jumping and balancing. (Although both jumping and balancing require whole body activation, it is definitely arguable that they are similar.) It was neither based on the design of the product; the used insole can apply the vibration from 0 to about 200% of the sensory threshold of most participants with fine resolution.

So, let us explain the rationale behind 130% more clearly in this response. First, the main motivation of the study was the recently observed “sensory-to-motor overflow.” Thus, we aimed to apply detectable sensory stimulation rather than sub-threshold vibration; we needed to apply vibration with the amplitude of 110, 120, 130, 140%, etc. of the threshold. Second, we aimed to investigate whether the additional detectable tactile stimulation can increase the motor performance under maximal effort. Among various motor tasks that require maximal effort, we chose jump, considering the convenience and reliability of the measure of the motor output. However, unfortunately, we could not find any similar previous study that investigated the effect of vibration amplitude on “jump” performance; this fact also makes this study as a unique contribution to this field. The only study that investigated the effect of various amplitudes of detectable vibration on motor task was what we cited, so we simply consulted the “best available” reference and chose 130%, which had been reported as the most effective level of the stimulus. So, the value of 130% was not based on either similarity between jumping and balancing (if any) or the design of the used insole. It was determined based on (limited) available data and reference. Considering reviewer’s comment, we revised the manuscript to clarify this (lines 371-376).

Despite this rationale, it is never guaranteed that our choice of 130% is the most effective level; rather, it is highly plausible that optimal parameters differ across individuals. In this initial study, we only demonstrated that some detectable vibration, a certain level of one kind of additional sensory input, can increase the motor performance under maximal effort. Tuning of the parameter of such stimulation requires much more future work. Considering reviewer’s comment, we revised the manuscript to further clarify this limitation and the necessary future work (lines 379-380).
